# Modified robotic-assisted laparoscopic pyeloplasty in children for ureteropelvic junction obstruction with long proximal ureteral stricture: The “double-flap” technique

**DOI:** 10.3389/fped.2022.964147

**Published:** 2022-10-14

**Authors:** Ce Han, Lifei Ma, Pin Li, Jia’nan Wang, Xiaoguang Zhou, Tian Tao, Hualin Cao, Yuandong Tao, Yunjie Yang, Yang Zhao, Weiwei Zhu, Tao Guo, Xuexue Lyu, Ran Zhuo, Huixia Zhou

**Affiliations:** ^1^Department of Pediatric Urology, Department of Senior Pediatrics, Chinese PLA General Hospital, Beijing, China; ^2^Department of Pediatric Urology, Bayi Children’s Hospital Affiliated to the Seventh Medical Center of PLA General Hospital, Beijing, China; ^3^Medical School of Chinese PLA, Beijing, China; ^4^Surgical Intensive Care Unit, The Second Medical Center of PLA General Hospital, Beijing, China; ^5^Department of Urology, Nanxi Shan Hospital of Guangxi Zhuang Autonomous Region, Guilin, China; ^6^Department of Urology, Southern Medical University Affiliated Nanhai Hospital, Foshan, China

**Keywords:** modified robotic-assisted laparoscopic pyeloplasty (MRALP), hydronephrosis, long proximal ureteral stricture, pediatrics, renal pelvic flaps

## Abstract

**Objective:**

The objective of this study is to introduce a novel technique of robotic-assisted laparoscopic pyeloplasty (RALP) for ureteropelvic junction obstruction (UPJO) with long proximal ureteral stricture in children.

**Materials and methods:**

Clinical information on patients who underwent a modified RALP between July 2018 and May 2019 in our center was collected retrospectively. Our surgical modifications mainly include “double-flap” tailoring of the renal pelvis and anastomosis of spatulate ureter with the double-flap. Demographic, perioperative, postoperative, and follow-up information was recorded in detail.

**Results:**

A total of 13 patients were included in the study. All the patients underwent a modified RALP without conversion to open surgery. They were followed up with a median time of 36 months. The anteroposterior diameter of the renal pelvis was 1.19 ± 0.21 at 6 months after the surgery, which was significantly lower than that on admission (3.93 ± 0.79). The split renal function of the children was also significantly improved from 0.37 ± 0.05) to 0.46 ± 0.02 at 6 months after surgery (*p* < 0.05). The diuretic renography revealed that all the patients have a T1/2 time less than 20 min postoperatively. The children were in good condition during the follow-up period.

**Conclusions:**

Modified RALP is an effective surgical treatment for children with UPJO with long proximal ureteral stricture. The success rate of this modification has been preliminarily confirmed.

## Introduction

Hydronephrosis, defined as dilation of the renal pelvis and/or calycesis, is a common congenital abnormality in the urological system of children (1). Ureteropelvic junction obstruction (UPJO) is the main cause of hydronephrosis (2). An untreated disease can result in renal impairment, making effective management crucial. The gold standard surgical treatment for UPJO is the Anderson-Hynes dismembered pyeloplasty technique described by Anderson and Hynes (3, 4). Owing to the potential high tension of anastomosis, conventional dismembered pyeloplasty is a relative contraindication for patients with complex conditions, especially for long proximal ureteral strictures (5).

Several surgical techniques have been adopted to address the considerable challenge of long proximal ureteral stenosis. Renal autotransplantation (6) or intestinal replacement (7) is a possible solution but always results in vascular or bowel complications. The appendix (8), oral mucosa (9), and renal pelvis (10) have also been proposed to manage the complex problem. Among them, the renal pelvis might be the best option, as it shares the same urothelium with the ureter (11). In this article, we introduced our “double-flap” technique and reported our preliminary experience.

## Materials and methods

### Patients

**FIGURE 1:**
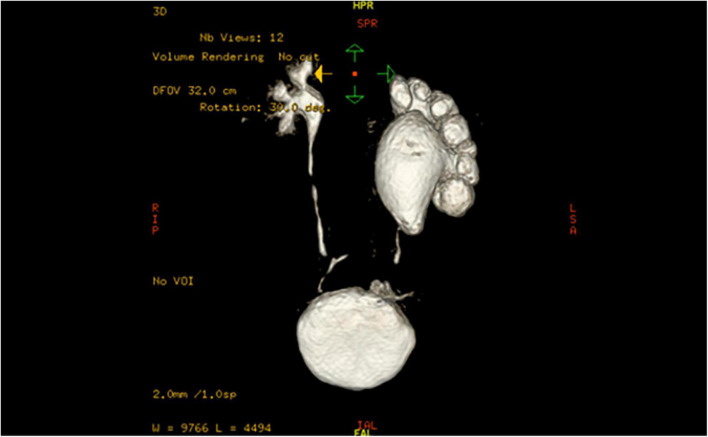
Magnetic resonance urography (MRU) results of the patients.

This study included children who underwent a modified robotic-assisted laparoscopic pyeloplasty (MRALP) in our center from July 2018 to May 2019. The indication for surgery was long proximal ureteral stricture (> 2 cm), which was not suitable for traditional end-to-end anastomosis. In addition, all patients should satisfy one of the following criteria before operation: ultrasonography screening showed that the separation of the anteroposterior diameter (APD) of the renal pelvis was > 3 cm; symptomatic hydronephrosis (flank pain, recurrent urinary tract infection, fever, and hematuria), diuretic renal radionuclide scan showing obstruction, and T1/2 > 20 min. Children with vesicoureteral reflux, congenital megaureters, and posterior urethral valve or other structural anomalies were excluded from the study. All the patients underwent ultrasonography, magnetic resonance urography (Figure 1), voiding cystourethrography (VCUG), and 99 mTc-mercaptoacetyltriglycine diuretic renography before surgery. The precise stricture length was measured intraoperatively.

Postoperative complications were evaluated with the Clavien–Dindo classification system. The study was approved by the Ethics Committee of the Seventh Medical Center of the PLA General Hospital (No. 2017-036). All the patients’ parents agreed to participate in the study and signed written consent forms.

### Surgical technique

**FIGURE 2:**
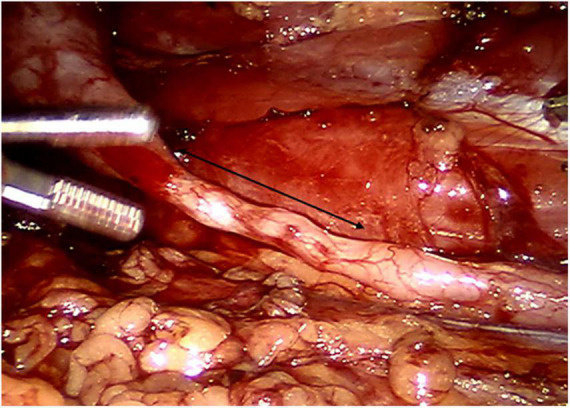
Measurement of the length of ureteral stricture (as indicated by the arrows).

**FIGURE 3:**
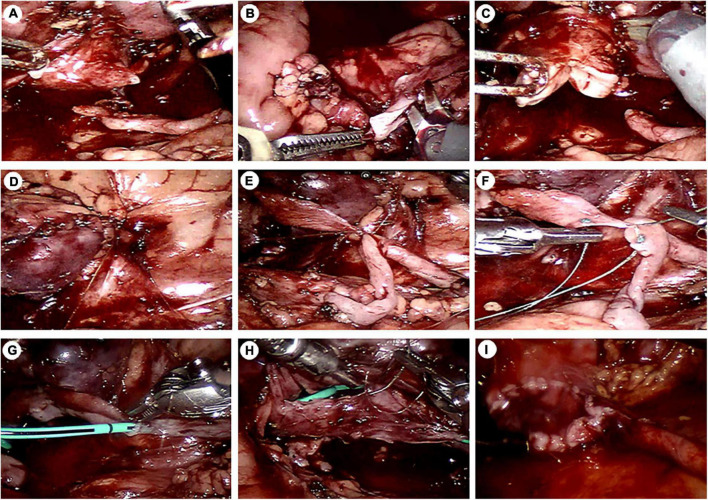
Intraoperative images. **(A)** The ureteropelvic junction was dismembered. **(B)** The ureter was cut longitudinally along the lateral edge of the ureter. **(C)** The renal pelvis was cut into double flaps. **(D)** The inferior pole of the kidney was fixed to the lateral abdominal wall. **(E)** The first stitch was placed between the lowest point of the inferior and the lowest point of the spatulate ureter. **(F)** The posterior wall was sutured continuously a using a traction suture. **(G)** A DJ stent was placed. **(H)** The anterior wall was anastomosed continuously. **(I)** The superior pelvis flap was sutured. Finally, the pelvis and the ureter together formed a “funnel.”

**FIGURE 4:**
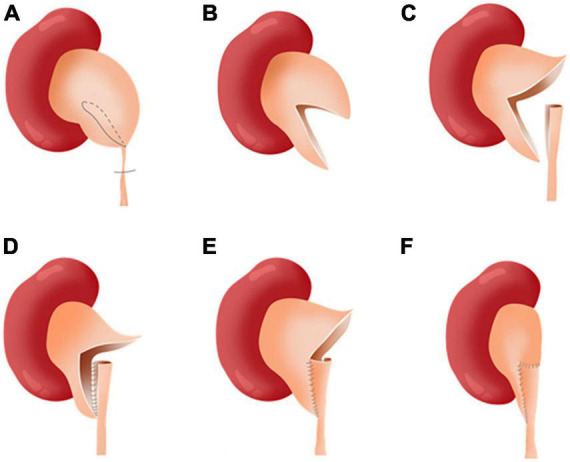
The “double-flap” technique. **(A)** Cut by line. **(B)** Pelvis double flaps. **(C)** Spatulate longitudinally along the lateral edge of the ureter. **(D)** Continuous posterior wall anastomosis. **(E)** Continuous anterior wall anastomosis. **(F)** Continuous superior flap anastomosis.

After general anesthesia, the child was placed 45–60 degrees from the horizontal direction (12). The trocar position has been described in an article previously published by our team (13). After the stricture ureter was located, we measured the length of the segment with a silk thread (Figure 2). Then we spatulated the ureter longitudinally along the lateral wall across the stricture segment of ureter. The length of spatulated ureter exceeded the stenotic ureter by 1 cm. Then, we incised the renal pelvis from the ureteropelvic junction to the lower middle third of the renal hilus. The ureter pelvis was cut into double flaps (Figures 3, 4). The inferior pelvis flap was trimmed to a tongue shape, and the first stitch was sutured between the inferior flap apex and the lowest corner of the spatulate ureter with a 6-0 monocryl suture. Then, a continuous suture was conducted between the posterior edge of the flap and the ureter. After a double-J stent was placed anterogradely, the superior pelvis flap was used to cover the defect area and anastomose continuously.

### Postoperative management

The double-J stent was removed under cystoscopy 6∼8 weeks after the operation. All the patients were followed up by ultrasonography 3, 6, 12, and 24 months after the operation. Six months after the operation, all the children underwent radionuclide and diuretic renography examinations.

### Statistical analysis

All data were analyzed with the SPSS 26.0 statistical software. The data was expressed as median [interquartile range (IQR)] or median (range). The preoperative and postoperative data were compared by Student *t*-test and Fisher exact test. *P* < 0.05 was considered a significant difference.

## Results

A total of thirteen patients were included in this study. All the operations were successfully completed without conversion to open surgery. The baseline clinical data of all the included patients are shown in Table 1. The perioperative information and postoperative complications are summarized in Table 2. The preoperative examination indices and postoperative follow-up of the children are statistically analyzed in Table 3. In this study, eleven boys and two girls were included, with a median age of 5 years. There were nine left side hydronephrosis patients and four right side hydronephrosis patients. Seven of the children experienced flank pain. Four children were observed because of prenatal hydronephrosis, which gradually deteriorated. One child had hematuria and one had fever due to urinary tract infection. The median length of ureteral stenosis was 2.5 cm (range 2–4 cm). Two patients had Clavien-Dindo grade II complications after the operation (intravenous antibiotic treatment for fever due to infection), but none of them had Clavien-Dindo III or IV complications. The children were followed for a median of 36 months. The APD of the renal pelvis at 6 months after the operation (1.19 ± 0.21) decreased significantly compared with that before the operation (3.93 ± 0.79) (*p* < 0.05). The split renal function also improved from (0.37 ± 0.05) to (0.5 ± 0.02) 6 months after the operation. The diuretic renography revealed that all the patients have a T1/2 time of less than 20 min at the same time.

**TABLE 1:** Demographic and clinical data of the patients.

Description	Value
Patients, n	13
Gender (male/female),n	11/2
Age (yr), median (IQR)	5 (4–9)
Affected side (left/right), n	9/4
Symptoms (flank pain/recurrent fever /hematuria/asymptomatic)	7/1/1/4
APD (mm), median (IQR)	4 (3.2–4.5)
Split renal function, median (IQR)	0.39 (0.37–0.40)
Renography T1/2 > 20 min	13

**TABLE 2:** Perioperative outcomes.

Description	Value
Operation time (min) median (IQR)	140 (125–160)
Estimated blood loss (ml),median (IQR)	15 (15–20)
Stricture length, cm, median (range)	2.5 (2–4)
Length of hospital after surgery, days, median (IQR)	6 (5–7)
Complications Clavien-Dindo	
I and II	2
III and IV	0

**TABLE 3:** Preoperative and follow-up characteristics.

Description	Pre-operation	6th month	12th month	24th month	*P*-value
APD (mm)	3.93 ± 0.79	1.19 ± 0.21	1.03 ± 0.13	0.98 ± 0.16	< 0.05
Split renal function	0.37 ± 0.05	0.46 ± 0.02	-	-	< 0.05
Renography T1/2 < 20 min	0	13	-	-	< 0.05

## Discussion

Treatment of UPJO with a concomitant long proximal ureteral stricture is often a dilemma for most urologists. Some experts continually explored the use of an autologous tissue to realize an alternative surgical technique, and some have achieved satisfactory results (9). Pelvic flaps, as a urothelial tissue, are one of the most preferred patch materials.

Culp and Dewierd reported the application of spiral and vertical flap technology in pyeloplasty in 1951 (14). Scardino and Prince affirmed the advantages of spiral and vertical flaps in reconstruction of proximal ureteral stenosis in 1953 (10). In 2002, Kaouk et al. reported the treatment of long ureteral stricture by laparoscopic dismembered pyeloplasty using the renal pelvis instead of the ureter. The length of the tubularized renal pelvis could be as long as 3 cm. When the pelvic tissue is adequate, adopting the pelvis to replace the ureter can be a choice (15). In 2007, Basiri et al. reported the application of a laparoscopic non-dismembered renal pelvis flap for treatment of patients with ureteral stenosis longer than 2 cm (16). In 2018, Sarihan et al. reported an open operation of reverse tubularized pelvis flap method for treatment of long ureteral stenosis. This study is the first report on patients aged 2–6 months. The study suggested that the reverse pelvic flap method is suitable for treatment of long segment UPJ obstruction in infants (17).

However, the conventional treatment has several limitations. Non-dismembered flap pyeloplasty cannot excise the obliterative, fibrotic, and non-peristaltic ureter. This situation leads to a reconstructed ureter lacking peristaltic function (18). For patients with poorly dilated renal pelvis, traditional dismembered pyeloplasty is not suitable because of lack of adequate renal pelvis tissue. The tubularized flap technique has the potential risks of restenosis and ischemia (19). Absorbing a previous experience, we modified the traditional pyeloplasty technique and developed the “double-flap” technique. This technique is quite suitable for extra renal pelvis, but it can also be used in patients with normal or small-sized renal pelvis. For intrarenal pelvis, this technique should not be applied.

This is the first study to conduct robotic-assisted laparoscopy to replace a part of the ureteral tissue with double renal pelvis flaps for treatment of UPJO with proximal ureteral stricture in children. Currently, surgical treatment has evolved from an open era to a minimally invasive era. Peters et al. first reported laparoscopic pyeloplasty in children in 1995 (20), and Gettman et al. first reported robotic pyeloplasty in 9 children in 2002 (21). Robotic and laparoscopic surgeries have achieved the same success rate as open surgery, with minimally invasive treatment, rapid postoperative recovery, and mild postoperative pain symptoms (22).

Based on our encouraging initial experience and clinical confirmation, our technical modifications proved reasonable. First, it avoids excessive pulling of the ureter. After cutting off the fibrotic and atresia ureter, the spatulated ureter can be anastomosed with pelvic flap at the horizontal level to avoid tension anastomoses caused by excessive pulling of the ureter that can result in ureteral scar formation and urinary leakage. In addition, the onlay anastomosis technique for the ureter and renal pelvis flap cannot only ensure good peristaltic function of the new ureter but also broaden the diameter of the upper ureter and form “a funnel” shape gradually tending to the normal diameter.

These key surgical procedures can help avoid postoperative restenosis. Moreover, traction sutures are used for fine operations to avoid the tissue ischemia caused by excessive clamping of the tissue (23). This method can also effectively avoid an unsmooth inner surface of the ureter lumen and excessive scar or polyp formation after surgery. In the anastomotic process, 6-0 monocryl is used for continuous suture, which can prevent the occurrence of postoperative urine leakage, and the scar reaction of anastomosis was minimal. In addition, to ensure blood supply to the ureter, the adipose tissue around the anastomotic ureter should be cleaned as much as possible to prevent the tissue from embedding into the anastomotic orifice and forming small polyps, which will adversely affect healing. Finally, we can attach the purpose that is to completely remove the diseased tissue, alleviate the patient’s symptoms, and protect the patient’s renal function.

This study had several limitations: the retrospective design has some bias when collecting the data. Moreover, this was a single-center study. Further validation of multicenter studies should be considered. This study included 13 patients, which is quite a small sample size. The effectiveness and advantages/disadvantages of this technology should be further verified by increasing the sample size.

## Conclusion

Our modified robotic pyeloplasty could be a practical and effective treatment option with high success rate for UPJO with long proximal ureteral stricture. However, a larger sample size, longer follow-up, and further controlled prospective studies are necessary to evaluate its safety and efficacy.

## Data availability statement

The original contributions presented in this study are included in the article/supplementary material, further inquiries can be directed to the corresponding author/s.

## Ethics statement

The studies involving human participants were reviewed and approved by the Ethics Committee of the Seventh Medical Center of the PLA General Hospital (No. 2017-036). Written informed consent to participate in this study was provided by the participants’ legal guardian/next of kin. Written informed consent was obtained from the individual(s), and minor(s)’ legal guardian/next of kin, for the publication of any potentially identifiable images or data included in this article.

## Author contributions

CH and PL: conception and design. HZ: administrative support. LM, XZ, and TT: provision of study materials or patients. WZ, YZ, and TG: collection and assembly of data. RZ and XL: data analysis and interpretation. All authors wrote the manuscript and approved the final version of the manuscript.
